# Interdisciplinary Management of Maxillary Midline Diastema With a Papillary Penetrating Labial Frenum Using Orthodontic Therapy and Diode Laser-Assisted Frenectomy: A Case Report

**DOI:** 10.7759/cureus.115694

**Published:** 2026-09-02

**Authors:** Shashank K, Barath Annadurai, Roopavathi KM, Praveen B Kudva

**Affiliations:** 1 Periodontology and Implantology, Sri Siddhartha Dental College & Hospital, Tumkur, IND; 2 Orthodontics and Dentofacial Orthopedics, Sri Siddhartha Dental College & Hospital, Tumkur, IND

**Keywords:** diode laser, frenectomy, interdisciplinary management, maxillary midline diastema, orthodontic treatment, papillary penetrating labial frenum, treatment sequencing

## Abstract

Maxillary midline diastema is a common esthetic concern with a multifactorial etiology that includes aberrant maxillary labial frenum attachment. A papillary penetrating labial frenum may contribute to the persistence of a maxillary midline diastema and may warrant consideration during treatment planning. We report a 19-year-old woman with a maxillary midline diastema associated with a papillary penetrating labial frenum. Comprehensive fixed orthodontic treatment was completed over 16 months, achieving closure of the diastema. Because the aberrant frenum persisted after space closure, a 980 nm diode laser-assisted frenectomy was performed under local anesthesia (pulsed mode, 3 W). The procedure was completed with minimal intraoperative bleeding, good hemostasis, and without sutures, and postoperative healing was uneventful, with satisfactory soft tissue maturation at the one- and three-month follow-up visits, after which a bonded lingual retainer was placed. The patient reported minimal postoperative discomfort and was satisfied with the overall esthetic result; no standardized pain or satisfaction scale was used. This case illustrates the clinical value of interdisciplinary sequencing, with orthodontic approximation first, followed by frenectomy before definitive retention, in a patient with a persistent papillary penetrating frenum. Within the limits of a single case with short follow-up, diode laser-assisted frenectomy may serve as a useful minimally invasive adjunct to orthodontic treatment in appropriately selected patients.

## Introduction

Maxillary midline diastema is a common esthetic condition characterized by a space between the maxillary central incisors. Although a transient midline diastema is a normal developmental stage during the mixed dentition, persistence of the space after eruption of the permanent dentition often necessitates clinical intervention because of its esthetic, functional, and psychosocial implications [1]. A visible diastema may adversely affect smile harmony and self-confidence and is one of the more frequent reasons for seeking orthodontic treatment [2]. Successful management depends not only on achieving space closure but also on identifying and appropriately addressing underlying etiological factors that may contribute to treatment stability.

The etiology of maxillary midline diastema is multifactorial and includes tooth-size discrepancies, congenital absence of teeth, supernumerary teeth, oral habits, periodontal conditions, and aberrant maxillary labial frenum attachments [1]. A papillary penetrating labial frenum has been associated with maxillary midline diastema and may contribute to persistence of the space in selected patients because extension of frenal fibers into the interdental papilla may interfere with approximation of the central incisors [3,4]. However, the available literature primarily supports an association between aberrant frenal morphology and the presence or persistence of maxillary midline diastema, rather than establishing the frenum as an independent predictor of post-treatment relapse [3,4]. Careful clinical assessment of the frenum is therefore an essential component of treatment planning in patients presenting with a persistent midline diastema [3].

Orthodontic therapy remains the primary treatment modality for achieving closure of the diastema. When an aberrant frenal attachment is identified, management of the associated soft tissue may also be required. The timing of frenectomy in relation to orthodontic space closure is an important consideration, with different treatment sequences described in the literature. Orthodontic closure before frenectomy is one approach used in selected cases, after which the need for soft-tissue intervention can be reassessed based on the residual frenal attachment and clinical findings [3]. In the present case, frenectomy was performed after orthodontic closure and before definitive retention.

Advances in laser technology have expanded the role of minimally invasive procedures in periodontal and oral soft tissue surgery. Among the available systems, diode lasers are widely used for frenectomy because of their affinity for soft tissues, enabling precise tissue ablation with effective hemostasis [5,6]. Laser-assisted frenectomy has been associated with reduced intraoperative bleeding and postoperative discomfort compared with conventional scalpel techniques, while studies of diode lasers, including 980 nm systems, have reported favorable soft-tissue healing and postoperative outcomes [6-8].

Although orthodontic space closure and laser-assisted frenectomy are well-established interventions, the appropriate sequencing of these procedures may vary according to the clinical presentation, particularly regarding the timing of frenectomy in relation to orthodontic space closure and definitive retention. This case report describes the interdisciplinary management of a 19-year-old woman with a maxillary midline diastema associated with a papillary penetrating labial frenum, in whom orthodontic approximation was completed before frenectomy and definitive retention. The case highlights the importance of reassessing the frenum after orthodontic space closure and tailoring the timing of soft-tissue intervention to the clinical findings.

## Case presentation

A 19-year-old woman presented to the Department of Orthodontics with a chief complaint of a persistent space between her upper front teeth, which negatively affected her smile esthetics and self-confidence. Her medical and dental histories were noncontributory, with no relevant systemic conditions or deleterious oral habits reported. Clinical examination revealed a persistent maxillary midline diastema between the maxillary central incisors (teeth 11 and 21), which measured 3.5 mm at presentation and was associated with a papillary penetrating maxillary labial frenum (Figure 1).

**Figure 1:**
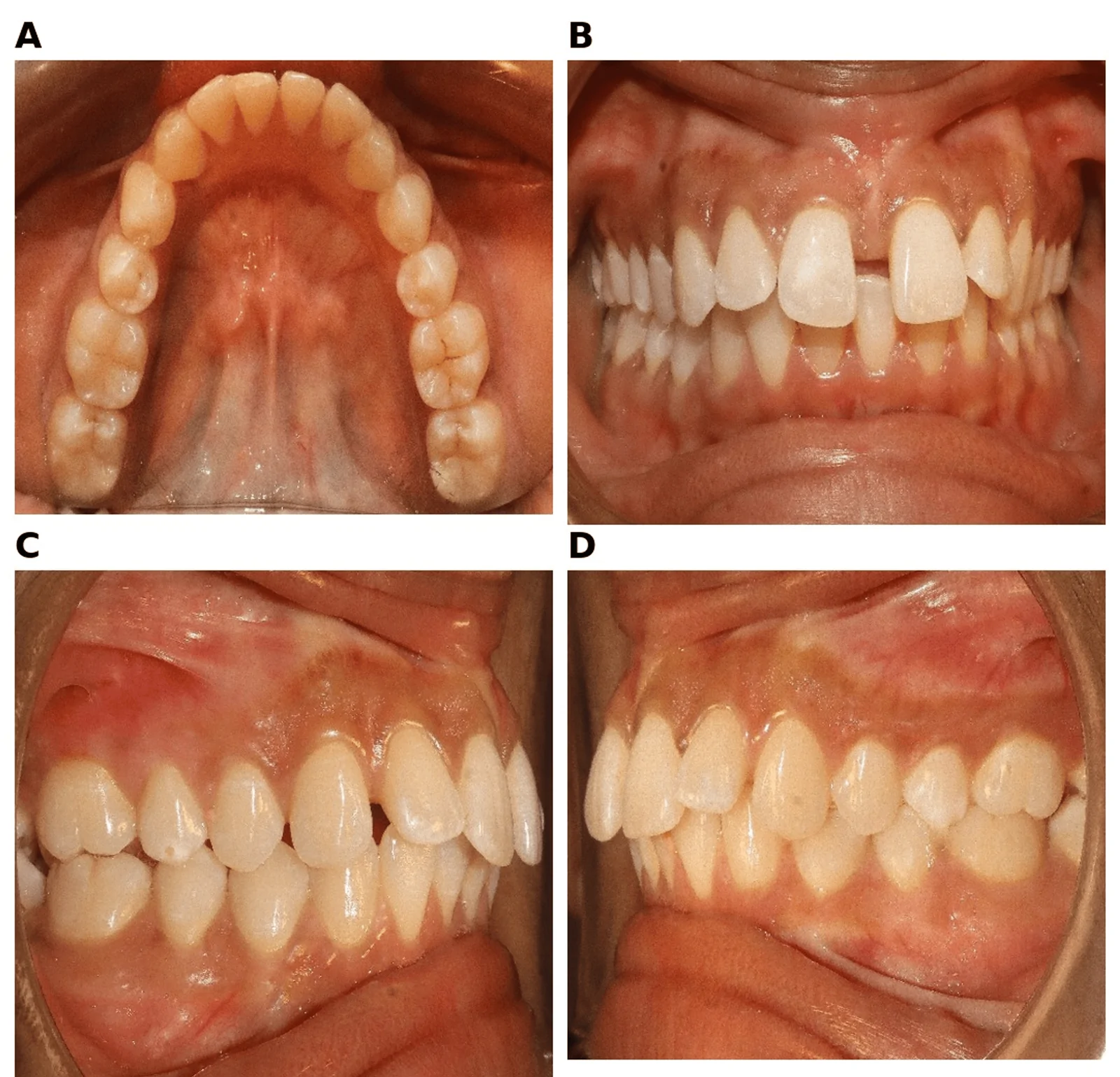
Preoperative intraoral photographs (A) Maxillary occlusal view; (B) frontal view demonstrating the 3.5 mm maxillary midline diastema between teeth 11 and 21 with a papillary penetrating labial frenum; (C) right lateral view; (D) left lateral view.

Clinically, the maxillary labial frenum was characterized as papillary penetrating based on the extension of the frenal attachment through the interdental papilla, and a positive blanching test was also observed. As part of the routine orthodontic assessment, a pretreatment panoramic radiograph (orthopantomogram) was obtained and revealed no significant dental or osseous abnormality (Figure 2).

**Figure 2:**
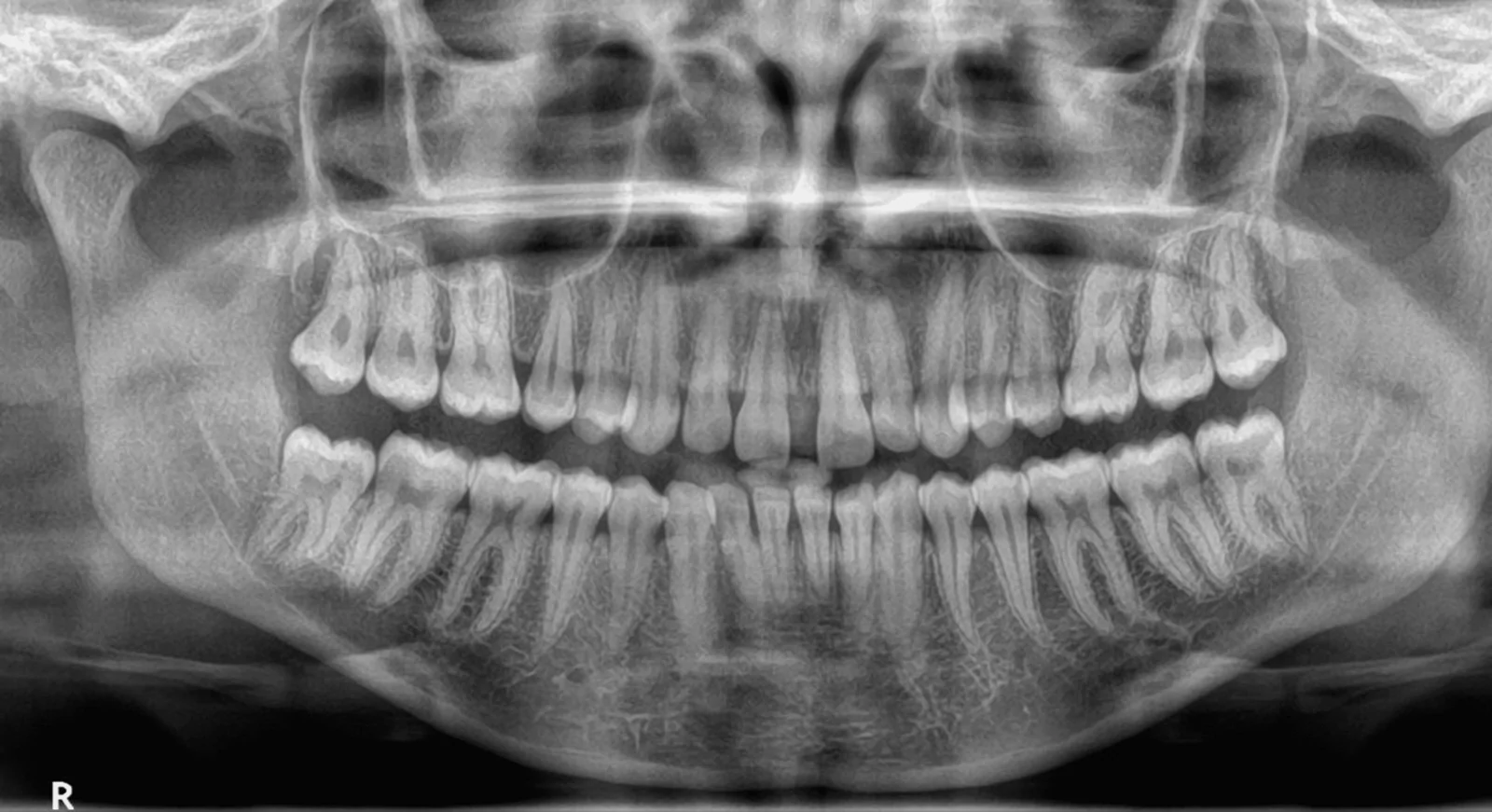
Pretreatment orthopantomogram Absence of an obvious supernumerary tooth, missing permanent tooth, or other significant dental or osseous abnormality associated with the maxillary midline diastema.

The clinical and radiographic assessment did not identify an obvious supernumerary tooth, missing permanent tooth, or other significant dental or osseous abnormality that could account for the diastema. Based on the clinical and radiographic findings, comprehensive fixed orthodontic treatment was planned to align the dental arches and close the maxillary midline diastema.

Comprehensive fixed orthodontic treatment was performed using a 0.022-inch slot preadjusted edgewise appliance. Initial alignment and leveling were achieved with sequential nickel-titanium archwires, followed by stainless-steel working archwires. The 3.5-mm maxillary midline diastema was progressively closed using an elastic chain with controlled mesial movement of teeth 11 and 21. No interproximal reduction was performed. Treatment was completed over 16 months, resulting in complete closure of the diastema with a final interincisal space of 0 mm and improved smile esthetics (Figure 3).

**Figure 3:**
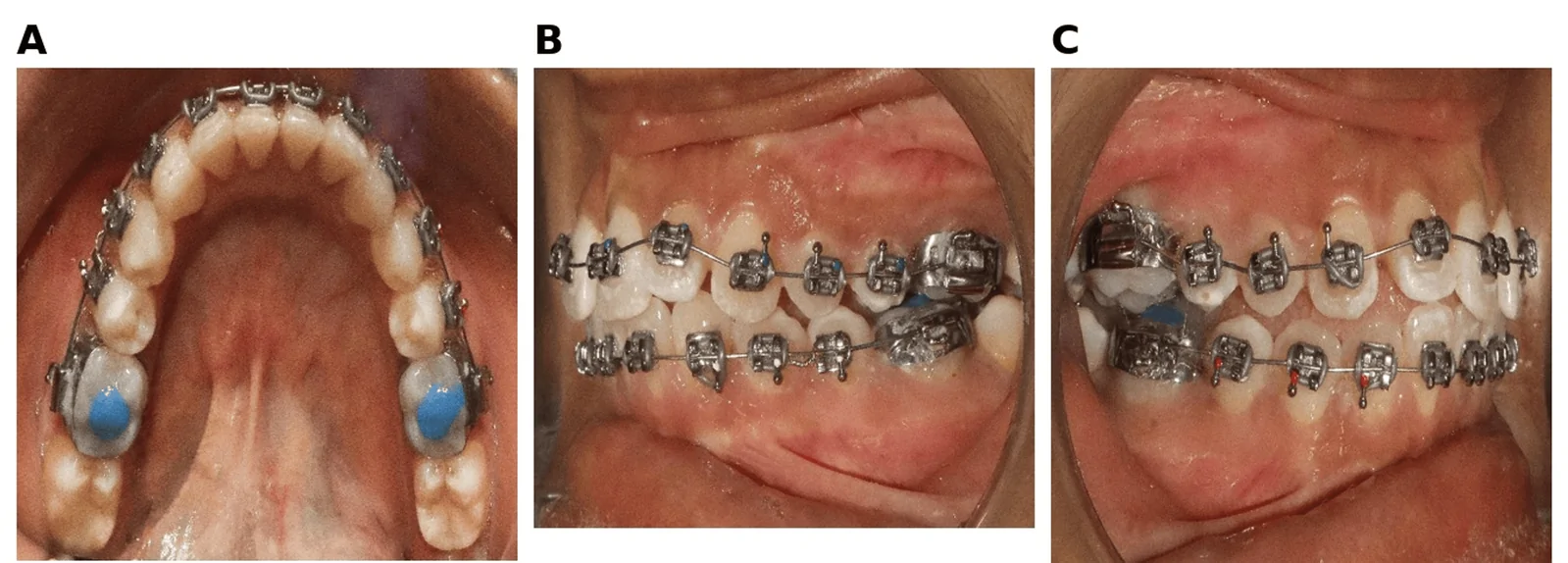
Orthodontic space closure before frenectomy (A) Maxillary occlusal view with the fixed appliance in place; (B) frontal view during active approximation of teeth 11 and 21; (C) frontal view demonstrating complete closure of the maxillary midline diastema before frenectomy, with persistence of the papillary penetrating frenum.

Following completion of orthodontic treatment, clinical reassessment confirmed persistence of the papillary penetrating labial frenum despite complete approximation of the maxillary central incisors. Considering the persistence of the aberrant frenal attachment after orthodontic space closure, diode laser-assisted frenectomy was planned before placement of the permanent retainer.

Written informed consent was obtained before the procedure. Under local anesthesia using 2% lidocaine with 1:100,000 epinephrine, frenectomy was performed using a 980-nm diode laser (Woodpecker LX16; Guilin Woodpecker Medical Instrument Co., Ltd., Guilin, China) operated in pulsed mode at an output power of 3 W, using a 400-µm fiber with an initiated tip and an application time of 45 seconds. The frenal tissue was carefully engaged and excised with controlled laser application until complete release of the fibrous attachment was achieved. In this patient, the procedure resulted in good intraoperative hemostasis, with minimal clinically observed bleeding. Owing to the coagulative properties of the diode laser, sutures were not required. A Coe-Pak periodontal dressing (GC America Inc., Alsip, IL, USA) was placed over the surgical site, and a postoperative analgesic (ibuprofen 400 mg every eight hours for three days, as needed) was prescribed. The laser application, immediate postoperative surgical site, and placement of the Coe-Pak periodontal dressing are shown in Figure 4.

**Figure 4:**
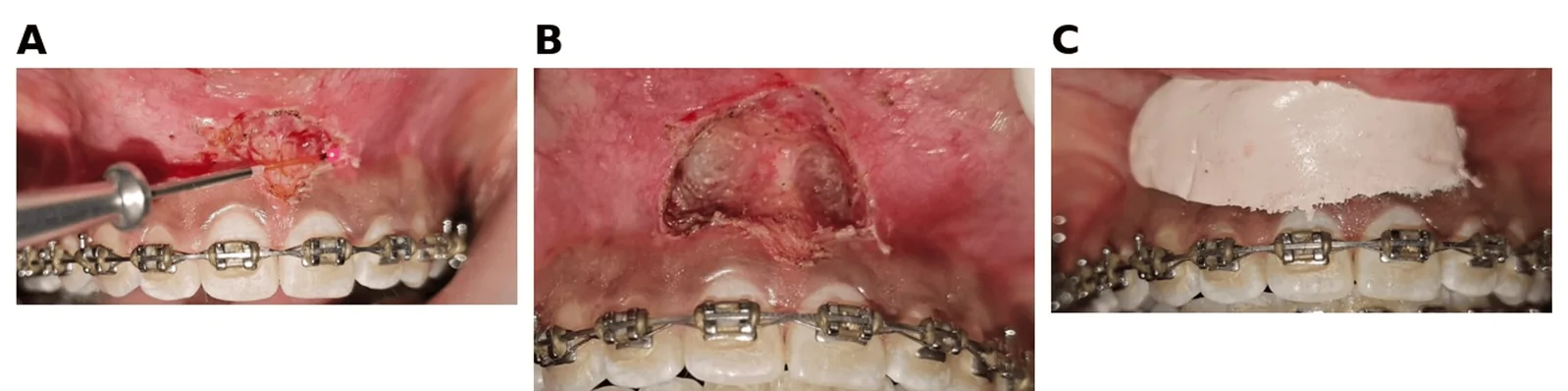
Diode laser-assisted frenectomy (A) Application of the 980 nm diode laser to the papillary penetrating labial frenum; (B) immediate postoperative appearance demonstrating excision of the frenal attachment and good hemostasis; (C) Coe-Pak periodontal dressing placed over the surgical site.

At the one-month follow-up, the surgical site showed clinically satisfactory healing, with healthy soft tissue architecture, adequate vestibular depth, and no clinical evidence of infection, excessive scar formation, or tissue contraction (Figure 5).

**Figure 5:**
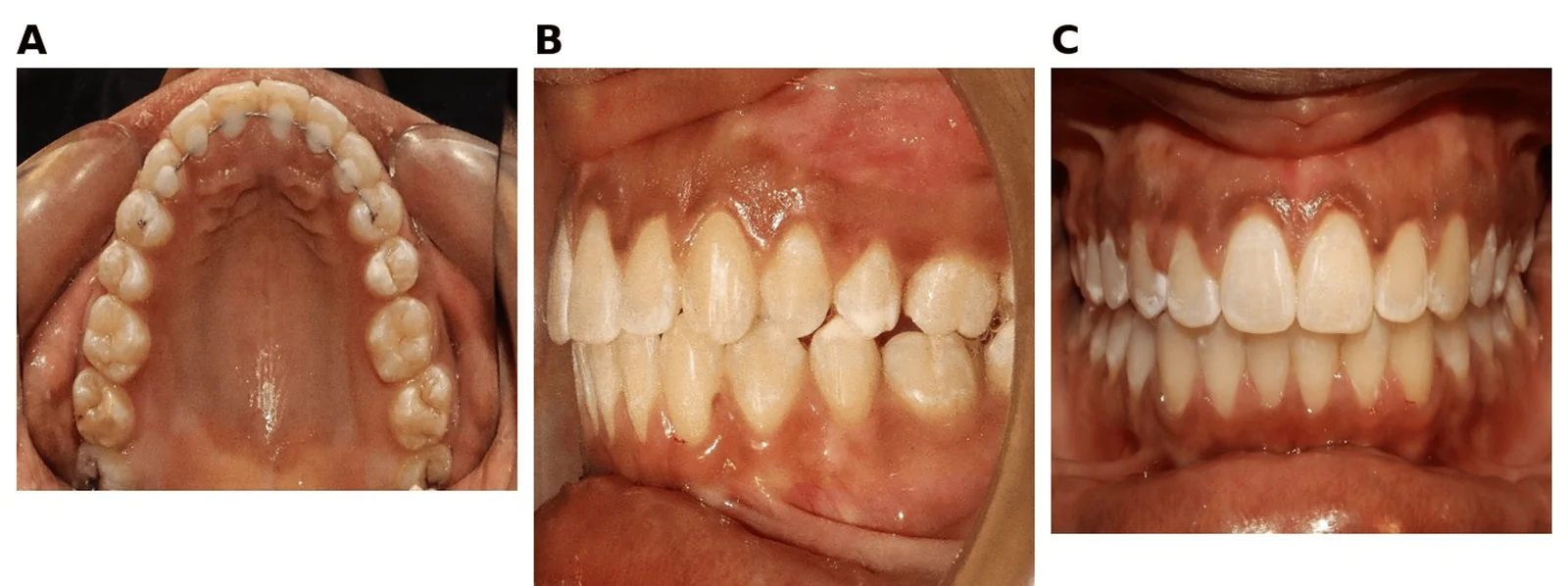
Clinical appearance at the one-month follow-up (A) Maxillary occlusal view; (B) lateral view; (C) frontal view demonstrating satisfactory soft tissue healing and maintained closure of the diastema.

At the three-month follow-up, continued soft tissue maturation was observed, and the orthodontically achieved diastema closure was maintained at this time point (Figure 6).

**Figure 6:**
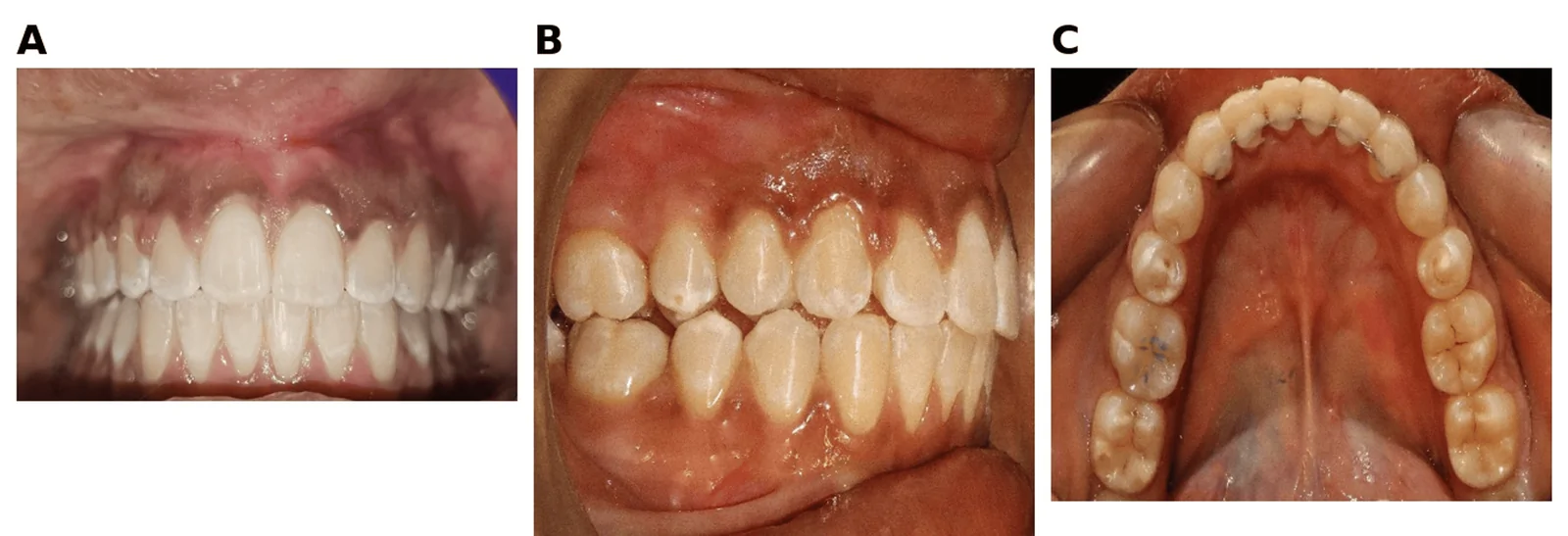
Clinical appearance at the three-month follow-up (A) Frontal view demonstrating complete soft tissue healing and maintenance of the orthodontically achieved diastema closure at three months; (B) additional frontal view; (C) maxillary occlusal view.

This follow-up period allowed assessment of early soft tissue healing but was insufficient to determine long-term orthodontic stability or exclude future diastema relapse. A bonded lingual retainer was subsequently placed as part of the retention protocol. The patient reported minimal postoperative discomfort and was satisfied with the overall esthetic result. A summary of the clinical timeline is presented in Table 1.

**Table 1:** Clinical timeline of the interdisciplinary treatment sequence Tooth numbering follows the FDI two-digit system (11, maxillary right central incisor; 21, maxillary left central incisor). OPG, orthopantomogram; FDI, Fédération Dentaire Internationale

Timepoint	Clinical event/intervention
Baseline (day 0)	Persistent maxillary midline diastema (teeth 11, 21) with a papillary penetrating labial frenum; diagnostic records and a pre-treatment orthopantomogram (no significant dental or osseous abnormality).
0-16 months	Comprehensive fixed orthodontic therapy; progressive approximation of the central incisors and closure of the diastema.
End of orthodontic phase	Papillary penetrating frenum persisted despite complete space closure.
Frenectomy (pre-retention)	980 nm diode laser-assisted frenectomy (pulsed mode, 3 W) under local anesthesia; good hemostasis; no sutures; Coe-Pak dressing applied.
One-month follow-up	Satisfactory healing; adequate vestibular depth; no infection, scarring, or contraction.
Three-month follow-up	Continued soft tissue maturation; orthodontically achieved diastema closure maintained at three months
Post-three-month follow-up	Bonded lingual retainer placed as part of the retention protocol.

## Discussion

Maxillary midline diastema is a frequently encountered esthetic concern in clinical practice and may have a considerable psychological impact by influencing smile esthetics and self-confidence. Although a transient midline diastema is a physiological finding during the mixed dentition, persistence after eruption of the permanent dentition generally warrants comprehensive evaluation to identify the underlying etiological factors [1,2]. Accurate diagnosis is essential because long-term success depends not only on orthodontic space closure but also on addressing the potential local factors contributing to the persistence of the diastema.

The etiology of maxillary midline diastema is multifactorial and includes tooth-size discrepancies, congenital absence of teeth, supernumerary teeth, oral habits, periodontal conditions, and aberrant maxillary labial frenum attachments [1]. A papillary penetrating labial frenum has been associated with maxillary midline diastema and may contribute to persistence of the space in selected patients because extension of frenal fibers into the interdental papilla may interfere with approximation of the central incisors [3,4]. In the present case, clinical examination demonstrated a papillary penetrating maxillary labial frenum associated with a persistent midline diastema, while panoramic radiography revealed no significant dental or osseous abnormality, facilitating appropriate interdisciplinary treatment planning.

Orthodontic treatment remains the primary modality for correcting maxillary midline diastema through controlled approximation of the central incisors, but successful management requires consideration of the associated soft tissue when an aberrant frenum is present. The timing of frenectomy in relation to orthodontic space closure has been debated, with different treatment sequences described in the literature, and the optimal timing remains dependent on the individual clinical presentation and treatment objectives [3,8]. In the present case, frenectomy was performed after orthodontic closure, allowing assessment of the persistent frenal attachment before definitive retention.

Diode lasers are widely used for soft-tissue procedures such as frenectomy because their tissue interaction permits precise ablation with effective coagulation of small blood vessels, contributing to good intraoperative hemostasis [5,7]. Diode laser surgery is commonly associated with good intraoperative hemostasis, improved visualization of the surgical field, reduced postoperative discomfort, and favorable wound healing [6-8]. Compared with conventional scalpel techniques, laser-assisted procedures often eliminate the need for sutures and reduce tissue trauma, thereby improving patient comfort and simplifying postoperative care [7,8].

A comparative study evaluating conventional scalpel technique against Nd:YAG laser frenectomy similarly reported reduced intraoperative bleeding and more favorable healing with the laser-assisted approach, supporting the broader trend toward laser-assisted soft-tissue surgery [9].

In the present case, frenectomy was successfully performed using a 980 nm diode laser in pulsed mode at 3 W. The procedure was completed with minimal intraoperative bleeding and good hemostasis, and, owing to the coagulative properties of the diode laser, suturing was unnecessary; only a Coe-Pak periodontal dressing was placed to protect the surgical site. The patient reported minimal postoperative discomfort, and clinical examination demonstrated uneventful healing with satisfactory soft tissue maturation at both the one- and three-month follow-up visits. The clinical findings observed in this patient are broadly consistent with favorable outcomes reported in comparative and observational studies of 980 nm diode laser-assisted frenectomy [8,10]. However, the present case does not permit direct comparison with conventional techniques or attribution of the observed outcomes specifically to the diode laser.

More recent comparative evidence has also evaluated different diode laser wavelengths, including 980 nm, against conventional scalpel technique, further supporting the clinical use of laser-assisted approaches while emphasizing that outcomes may vary according to the laser parameters and surgical technique [8]. The findings of the present case parallel these observations, particularly with respect to intraoperative hemostasis, minimal postoperative discomfort, and satisfactory soft tissue healing.

Long-term stability after correction of a maxillary midline diastema depends on appropriate management of both dental and soft tissue factors. Orthodontic space closure may be sufficient in many patients with maxillary midline diastema, whereas frenectomy may be considered in selected patients when an aberrant frenal attachment persists after orthodontic approximation or is considered clinically relevant. Conversely, frenectomy alone does not address an underlying malocclusion requiring orthodontic correction. Thus, the need for frenectomy should be determined on the basis of the individual clinical findings and treatment objectives [3]. An appropriate retention protocol is important because maxillary midline diastema closure may be prone to relapse after orthodontic treatment [11]. In the present case, the diastema closure was maintained throughout the three-month follow-up, after which a bonded lingual retainer was placed to support the treatment outcome.

From a clinical perspective, the principal lesson of this case is the value of individualized interdisciplinary sequencing. In this patient, orthodontic approximation of the central incisors was completed first, followed by reassessment and frenectomy before definitive retention. This approach allowed the persistent frenal attachment to be evaluated after space closure and addressed before retention. In this patient, the 980 nm diode laser was used to remove the aberrant frenum, with good clinically observed hemostasis and minimal reported postoperative discomfort. These findings represent observations from this individual case and do not establish superiority of the diode laser over conventional frenectomy techniques [8,10].

Limitations

This report describes a single patient, and the three-month follow-up is insufficient to establish long-term stability of the orthodontic result or to exclude subsequent diastema relapse. Objective measures of intraoperative bleeding and validated patient-reported outcome measures, such as pain or satisfaction scales, were not recorded. Prospective comparative studies with larger samples, longer follow-up, and standardized outcome measures are required to compare diode laser-assisted frenectomy with conventional techniques and to evaluate long-term treatment stability [7,10]. In addition, the absence of a comparative treatment approach prevents attribution of the observed clinical outcomes specifically to the diode laser technique.

## Conclusions

Maxillary midline diastema associated with a papillary penetrating labial frenum requires careful diagnosis and individualized treatment planning. In the reported patient, orthodontic approximation of the central incisors was followed by 980 nm diode laser-assisted frenectomy before definitive retention, with satisfactory soft tissue healing and maintenance of diastema closure at the three-month follow-up. These findings represent the clinical outcome observed in this individual patient and do not establish the superiority or effectiveness of this treatment sequence or the 980 nm diode laser technique over alternative approaches. Longer follow-up and comparative studies are required to evaluate long-term orthodontic stability and the relative benefits of different frenectomy techniques.
